# Role of traditional healers in the management of severe malaria among children below five years of age: the case of Kilosa and Handeni Districts, Tanzania

**DOI:** 10.1186/1475-2875-5-58

**Published:** 2006-07-18

**Authors:** Emmanuel A Makundi, Hamisi M Malebo, Paulo Mhame, Andrew Y Kitua, Marian Warsame

**Affiliations:** 1 National Institute for Medical Research (NIMR), P O Box 9653 Dar es Salaam, Tanzania; 2 Division of International Health, Department of Public Health Sciences, Karolinska Institute, Sweden

## Abstract

**Background:**

The current malaria control strategy of WHO centres on early diagnosis and prompt treatment using effective drugs. Children with severe malaria are often brought late to health facilities and traditional health practitioners are said to be the main cause of treatment delay. In the context of the Rectal Artesunate Project in Tanzania, the role of traditional healers in the management of severe malaria in children was studied.

**Methodology:**

A community cross-sectional study was conducted in Kilosa and Handeni Districts, involving four villages selected on the basis of existing statistics on the number of traditional health practitioners involved in the management of severe malaria. A total of 41 traditional health practitioners were selected using the snowballing technique, whereby in-depth interviews were used to collect information. Eight Focus Group Discussions (FGDs) involving traditional health practitioners, caregivers and community leaders were carried out in each district.

**Results:**

Home management of fever involving sponging or washing with warm water at the household level, was widely practiced by caregivers. One important finding was that traditional health practitioners and mothers were not linking the local illness termed *degedege*, a prominent feature in severe malaria, to biomedically-defined malaria. The majority of mothers (75%) considered *degedege *to be caused by evil spirits.

The healing process was therefore organized in stages and failure to abide to the procedure could lead to relapse of *degedege*, which was believed to be caused by evil spirits. Treatment seeking was, therefore, a complex process and mothers would consult traditional health practitioners and modern health care providers, back and forth. Referrals to health facilities increased during the Rectal Artesunate Project, whereby project staff facilitated the process after traditional medical care with the provision of suppositories. This finding is challenging the common view that traditional healers are an important factor of delay for malaria treatment, they actually play a pivotal role by giving "bio-medically accepted first aid" which leads to reduction in body temperature hence increasing chances of survival for the child. Increasing the collaboration between traditional healers and modern health care providers was shown to improve the management of severe malaria in the studied areas.

**Interpretation and conclusion:**

Traditional health care is not necessarily a significant impediment or a delaying factor in the treatment of severe malaria. There is a need to foster training on the management of severe cases, periodically involving both traditional health practitioners and health workers to identify modalities of better collaboration.

## Introduction

Malaria is a major public health problem in Tanzania with about 16 million cases being reported annually in a population of 34.5 million [1]. The disease constitutes the leading cause of hospital attendance and admission as well as the leading cause of death for all ages in the country, the greatest burden being in children under five years of age. Estimate indicates that annual deaths due to malaria range from 100,000 to 125,000, out of which 70,000 to 80,000 are children under five years of age [2]. Malaria has remained one of the five major life-threatening childhood conditions, resulting in an annual death toll of more than one million of African children [3]. In the light of the current WHO strategy to promote early recognition and treatment of malaria, community understanding and specifically traditional healing practices for malaria have become important aspects of investigation. Previous studies have revealed that in African communities, convulsion due to malaria is attributed to different causes, such as leaving a child on cold earth [4], a bird-like spirit possessing a child [5-7] or cold spirit [8].

Biomedically, malaria is a well-defined disease, in which parasites of the genus *Plasmodium *are transmitted to humans through the bite of female mosquitoes belonging to the genus *Anopheles*. The disease is also known to be associated with fever, headache, chills, shivering, loss of appetite, vomiting, general body weakness and joint pains. Such a biomedical conceptual framework has continuously been challenged when the perspectives of the people in a community are concerned. In the socio-cultural context, traditional beliefs are the basis for the local definitions of health problems occurring in the community as to whether they are due to natural causes, spirits or bewitchment. Thus, treatment seeking for childhood malaria depends very much on the manifestations of the disease in which the conception of natural or unnatural disease will be derived. Such a local body of knowledge, developed as an interplay between biomedical and traditional beliefs and logics, has been reported to guide people in deciding on treatment routes, i.e. whether to consult traditional healers or health facilities or both systems at the same time [9].

In recent years, great emphasis has been placed in investigating socio-cultural factors that influence health-seeking behaviour for malaria and cause delay in attending health facilities [10]. An ethnographic study of childhood malaria in southeastern Tanzania revealed that individuals made a clear distinction between 'normal malaria' (*malaria ya kawaida*) and 'cerebral malaria' characterized by convulsions (*degedege*) [9]. Actually, a convulsed child in a health facility is highly likely to be taken to a traditional healer in preference to any other health service. It has been suggested that in chronic or fatal illnesses, when symptoms change abruptly or when treatment does not provide the expected results, suspicion of witchcraft or spirits can emerge [12,13].

In Tanzania, as in most sub-Saharan Africa, prompt case diagnosis and effective treatment is the main control strategy for malaria. The government has made deliberate and successful efforts to make health care services accessible to the majority of rural communities. However, this does not guarantee that all patients utilize the services when they fall ill. Since malaria may present in different forms like convulsions, altered consciousness and coma, community perception of underlying causes may differ. Consequently, many patients with these conditions turn to traditional healers prior to seeking modern health care resulting in delay in receiving effective treatment.

The Rectal Artesunate Project, which is currently taking place in Kilosa and Handeni districts in Tanzania, aims to identify and assist children with severe malaria, including convulsions and coma, in their referral to hospital where proper, equitable and adequate treatment is available. Therefore, it was thought necessary to find ways to identify patients with such conditions at an early stage and provide them with effective treatment. The exploratory study was planned to collect baseline information, which would be used as a basis for an intervention to find out ways of involving traditional healers in the management of severe malaria in children. A set of key topics regarding treatment seeking and management of severe malaria was investigated in the study areas. These are:

• **Community knowledge about malaria**: local definition, causes, symptoms, and more specifically, whether people link the symptoms and manifestations to malaria;

• **Treatment-seeking**: biomedical, traditional beliefs and logics which guide treatment-seeking paths;

• **Mode of acquisition of traditional healing knowledge**: spirits, apprenticeship

• **Traditional management of severe malaria**: home management, traditional healers management, referral system;

• **Collaboration of traditional healers and Rectal Artesunate Project**: willingness to collaborate, collaboration areas.

## Methods

### Study area

The study area comprises the districts of Kilosa in the Morogoro region of south-eastern and Handeni in the Tanga region of north-eastern Tanzania. The specific setting for this study is in a stable perennial malaria transmission belt. A multi-stage sampling procedure was used. The study districts were stratified into two strata. The stratification takes into account the geographical reasons namely northern and southern zones. Stratification also took into account the main ethnic divisions of the Kilosa district namely Sagara, Kaguru and Vidunda, and Zigua for the Handeni District. Each stratum was mapped to determine the number of traditional health practitioners.

A two-stage cluster sampling was conducted to select a sample of villages to be studied. The first stage involved a purposeful selection of one ward from each stratum in collaboration with Council Health Management Team (CHMT) members, taking into account the statistical number of traditional healers in the district. In stage two, two villages were randomly selected from the wards. Initially, traditional healers were identified, through key informants including local authorities, health workers from local health facilities, schoolteachers and elders in the community. A snowballing technique was employed to identify healers who, in turn, identified others. Sampling included traditional healers dealing with malaria case management. In a situation where the number was too large, a few were selected. More attention was given to traditional healers and Traditional Birth Attendants (TBAs) who treat among other diseases malaria/febrile illnesses to children five years of age and below. Mothers/caregivers of children under five years were selected in collaboration with health workers and TBAs in a situation where births occurred at home.

### Qualitative methods

Qualitative approaches including in-depth interviews and focus group interviews were applied to explore and describe management patterns of severe malaria in the study communities. In-depth interviews were conducted with the traditional healers and mothers/caregivers to get an insight on their knowledge of malaria, symptoms and health-seeking behaviour. Two focus group discussions (FGDs), one with traditional healers and another one with mothers/caregivers, 'significant others' and community elders were carried out in each village. These discussions aimed at getting views on childhood malaria and health seeking behaviour, local terminologies for mild and severe malaria and community views regarding the Rectal Artesunate Project in the area. FGD participants were usually selected on the basis of shared experiences and interests, knowledge of the subject under investigation and willingness to participate in the discussions [14]. Participants in the FGDs were selected in collaboration with key informants in the study areas. A key informant was considered any member of the study population who offered to inform or educate the researcher on a given subject of investigation [15,16]. The FGDs lasted between one and a half to two hours as recommended elsewhere in literature [15,16].

### Data analysis

Since most of data collected was qualitative in nature, text analysis software (Text-Base Beta (Centre for Qualitative Research, University of Aarhus, Denmark) was used. The codes permitted compilation of text segments of interest for thematic analysis. The STATA 7.0 (Stata Group) was used for analysis of quantitative information.

### Ethical considerations

A standard explanation and introduction was given out to each individual on the benefits of participating in the study before informed consent was sought. Individuals were asked whether they had any questions and whether they agreed to take part in the study. The study used qualitative methods (interviews and discussions) with minimal risks to the participants. Confidentiality of all study participants was assured. Ethics clearance was obtained from Medical Research Co-ordinating Committee (MRCC) of the National Institute for Medical Research (NIMR), Tanzania.

## Results

### Demographic characteristics

The socio-demographic profiles of the study groups are summarized in Table 1.

**Table 1:** Socio-demographic characteristics of respondents

**Characteristics**	**Mothers/caretakers**	**Traditional healers**
**No. participants**	84	41
**Gender**		
Females	41 (100%)	14 (34%)
Males	0 (0%)	27 (66%)
**Age (years)**		
15–19	2 (2.4%)	0 (0%)
20–29	46 (54.8%)	0 (0%)
30–39	30 (35.7%)	3 (7.3%)
40–49	6 (7.1%)	3 (7.3%)
50–59	0 (0%)	5 (12.2%)
60–69	0 (0%)	5 (12.2%)
70–79	0 (0%)	18 (44%)
80–89	0 (0%)	7 (17%)
**Educational level**		
None	24 (28.6%)	17 (41.5%)
Primary	57 (67.9%)	24 (58.5%)
Secondary	3 (3.5%)	0 (0%)
Above secondary	0 (0%)	0 (0%)

### Mode of acquisition of the traditional healing practice

The mode of acquisition of the traditional healing practice described by the interviewed healers fall into four major paths:

1. Training through apprenticeship

2. Inheritance from member of the family

3. Dreamed instructions from the ancestral spirits

4. Falling sick and become a healer on recovery

Most of the traditional healers interviewed (31 out of 41), indicated to have passed through a combination of routes 1, 2 and 3. All of the 31 were raised in families with traditional healers, who involved them in the healing process and they were familiar with the profession at the time they started to practice on their own [17]. All reported on the importance of the family environment of a traditional healer in the context of acquiring knowledge and experience by members of the family. Furthermore it was reported that, the entrance into practice through these routes is facilitated through training by an experienced healer or a family member, who decides when the apprentice is ready to become an independent healer or to take up the practice. Eight out of 41 of the traditional healers indicated to have been instructed through dreams by their ancestral spirits to take up the traditional healing practice. They were required to learn and observe traditional healing procedures as dictated by the spirits. Indeed, the ancestral spirits are considered to be supernaturally powerful and ignoring them is to welcome punishment to an individual and or her family. Of the 41 healers interviewed, two indicated to have become healers after unknown illness initiated by spirits. One 70-year old healer from Kilosa expressed it this way:

"I experienced unknown illness which took away my senses, then I ran away into the forest and lived in a cave for three years. After that duration, I came back into the village as a horrible creature with long hairs, nails and a stinking body. My relatives ran away and some of the courageous ones got hold of me and took me directly to one traditional healer for divination. The outcome of divination indicated that the ancestral spirits have already completed their training on me to become a healer, thus instructing my relatives to cut my hairs and nails and thereafter the traditional healer conducted a series of rituals on me. After that my senses came back and I have become a traditional healer for over 40 years now!"

A similar account was reported by Geissler et al [17] in a short portrait on how a traditional healer in Kilama area, Kilombero district became a healer following an illness.

The long time duration of practice ranging from 10 to 61 years in the surveyed traditional healers indicates their long-term experience in dealing with community health problems and the corresponding trust the people have bestowed upon them.

### Community knowledge about malaria

The results show that the two studied communities held good biomedical knowledge of febrile illnesses. Mothers identified three distinct types of febrile illnesses as mild malaria (*malaria ya kawaida*), severe malaria (*malaria kali*) and convulsions (*degedege*). Symptoms of "*malaria ya kawaida*" mentioned were ordinary fever, chills, body weakness, loss of appetite and vomiting, whereas; "*malaria kali*" was identified with high fever, shivering, loss of appetite, child becoming very weak and sleeping all the time; while "*degedege*" identified with high fever, loss of appetite, vomiting, rigid body and un-coordinated movements of the limbs and eyes.

In this study, respondents interviewed mainly attributed "*malaria ya kawaida*" and "*malaria kali*" to mosquitoes. Other mentioned causes were change of weather, bad air and polluted breast milk. Although the mentioned symptoms for the three conditions are related with some degrees of differences, in most cases mothers were not linking signs and symptoms of "*degedege*" to malaria. *Degedege *was believed to be caused by spirits, bird-like creature and a moth.

### Treatment-seeking

Interviews involving mothers, traditional healers and community leaders indicated that most mothers, traditional healers and leaders in the study communities were conversant with the signs and symptoms of malaria; a description given was consistent with the Western definition of mild malaria. Mothers also described a severe childhood illness called '*degedege*', consistent with convulsions. Most of the mothers could not associate this condition with malaria, believing it is caused by evil spirits and should be treated by traditional methods such as urinating on the sick child or fumigating with elephant dung smoke.

A 36-year old mother from Kilosa, participating in the discussion explained what she considered the cause of degedege;

*"Degedege is caused by bad spirits. In this realization, spirits that cause convulsion must be removed first so that Western and other medication can work in treating the child. That is why we start at the traditional healer for treatment of a convulsed child and later we take him or her to hospital"*.

Yet another traditional healer, 70 years old, from Handeni added during the discussion sessions:

*"Children are normally attacked by spirits which cause uncoordinated movement of eyes and limbs. The disease is better understood traditionally and the spirits easily and quickly leaves the child when urinated on, fumigated with elephant dung smoke and with other herbs as well as washing the convulsed child with herbal water. Mothers know this very well and that is why they bring convulsed children to us for treatment"*.

Traditional healers were considered as the most important primary source of treatment outside homes for this condition. A 30-year old mother from Kilosa put it this way:

*"When my child convulsed, I urinated on her and took her immediately to the traditional healer as the traditional healer is conversant with the treatment of evil spirits causing degedege. I could not take her to hospital as in the hospital they treat by injecting drugs, the practice which results to sudden death of children under such treatment"*.

Yet, another middle-aged mother gave her own experience:

*"When my child convulsed, I urinated on her and smoked elephant dung. She cried and passed stool. After that, I took her immediately to the traditional healer for herbs, which can completely prevent degedege. After that I took her to the dispensary for malaria treatment"*.

One 60-year old Traditional Birth Attendant (TBA), from Kilosa, substantiated further on the motivation to send convulsing children to traditional healers first, as injections were considered fatal for such condition as used in modern system.

*"It is because of fear and mistrust of Western medicine treatment which is carried out by use of injections, mothers decide to send convulsed children to traditional healers as possible survival is predictable compared to hospital treatment. I know if I take my convulsed child to hospital they may inject him and this practice is known to kill children. On this background, I avoid hospital treatment for degedege and what I normally do is to send the convulsed child to the traditional healer for treatment and then after that, send the child to hospital for check up and treatment of malaria fever"*.

A Ward Executive Officer (WEO) explaining his experience in the area further explains;

*"Mothers traditionally believe that degedege is caused by bad spirits. Based on this belief, they send convulsing children to traditional healers for treatment first and then to hospital"*.

The above accounts show that, in the study areas, treatment seeking for children suffering from malaria is a complex process, being a function of socio-cultural *milieu *in which people live. Generally, mothers would consult different healing resources at the same time starting with traditional healers and then modern care, back and forth.

### Traditional management of severe malaria

#### Home management

The most common way practiced by mothers for treating a convulsing child at home included sponging or washing by cold water believed to reduce the temperature 22% (N = 84), placing the febrile child under bed and urinating on him/her especially in Kilosa District 12% (N = 84), elephant dung smoke infusion '*mavi ya tembo*' 8% and smearing herbs on the child body (10%). Others said they do nothing at home, instead resort to either traditional healers or health facilities 58% (N = 84). This is also indicated in other studies in Tanzania [18], which showed that home management of mild and severe malaria in Tanzania was a common practice.

One way of managing a child suffering from mild or severe malaria at home by mothers involved a mother urinating on the child '*mama kumkujolea mtoto wake*', which is believed to lower the temperature of the child, thus serving as 'First Aid'.

It follows, therefore, that home management of a mild or severe case of malaria is a common practice, and it is only after the observation of the outcome that a child is taken to another medical resource i.e. traditional healers or nearby health facility. The findings of this study indicate that mild or severe cases could be associated with various causes and establishing and negotiating diagnoses involved different types of specialists as well as what has been referred to as 'Therapy Management Group (TMG)' [19]. In many cases, it was found out that it was not the mother alone who made the decision on where to take a child when the situation deteriorates, but 'significant others' including the grandmother, aunt and sometimes a neighbour also played a vital role.

#### Traditional healers management

Traditional healers identified at least five stages through which a child suffering from severe malaria must pass during the healing process. It must be pointed out, however, that not all the healers would necessarily pass through the 5 stages, but the stages of diagnosis and treatment were considered paramount. Although there were some variations from one healer to another, generally the process involved the following stages:

##### 1^st ^Stage: Reception

This entailed warm welcome of the mother to the compound and greetings according to the local culture. At this session, the mother is required to explain the ailment of the child, when the ailment started and the steps taken by the mother so far to address it.

##### 2^nd ^Stage: Bathing/sponging the child to get the temperature down

This was a necessary second step involving certain forms of homecare, including bathing the child using grounded local herbs "*mwingajini*', sponging by using warm water and, in some cases, the mother of the child urinating on her child, '*mama kumkojolea mtoto wake*', in which the child is positioned under the bed and the mother over the bed, after the mattress has been removed.

##### 3^rd ^Stage: Diagnosis (Divination or reading the Quoran)

This stage involved several forms with the objectives of finding out the exact causes of the situation in hand. Most healers were herbalists-cum-diviners. Divination was done by ringing the bell '*kupiga kengele*' or reading the Quoran. The outcome of divination was to identify appropriate treatment or solution of the problem. It appeared that in five cases the mother needed to be treated first, so as to prepare for a smooth healing for the baby, in the belief that evil spirits possessing the mother are causing illness to her child. One 76-year old female, traditional healer in Mswaki, Handeni, who has 30 years of experience, expressed it this way:

"I must first of all find out if the mother is bewitched by evil spirits- that may cause the child illness, especially if the mother was not 'cleansed' from the spirits. Thereafter, I treat the child. In so doing I ensure that the bad spirits will not harm the child again through the mother."

The above account indicates, therefore, that diagnosis of the problem goes beyond the current problem afflicting the child to a broader societal causes of ailment especially if there is suspicion that evil sprits may be the root cause, as shown in other studies.

##### 4^th ^Stage: Treatment

The treatment procedure followed the divination or diagnostic process, where specific medicine or treatment would be known. The most common local herbs mentioned were katula mpama, kivumbasa (*Ocimum suave*), Mhasu, Mlwati, Mlama, Mkongodeka, Unganyiki, Mkwaju (*Tamarindus indica*), Bhangi (*Cannabis sativa*), Mlingo, Mvungaliza, Mdimbogo, Mbolo, Mkunde Kitimbilikwima (*Senna auriculata*), Lufyambo, Mhofya, Mningajini (*Cassia abbreviata*), Mnungu (*Zanthoxylum chalybeum*), Mninga (*Pterocarpus angolensis*), Mzizima, Mfiwi, Mbaazi (*Vigna unguiculata*), Mkwambekwambe, Mtunduru, Kibwamsongera, Fuzifuzi, Mhuwangulu, Turatura (*Solanum incanum*), Vitunguu Swaumu (*Allium spp*), Mwaraka, Kitako cha Nge, Msalaka (*Saraka Indica*), Lwenya, Msekela, Ngurukila, Mfunguo and Mkulagembe. These herbs were grounded and at times boiled then administered while using cold or boiled water. Some were given to the mother of child to be administered at home for three to five days. Generally, the treatment ranged from two to seven hours for healers who do not hospitalise the patients, and two to five days for those who do.

Findings from interviews with traditional healers indicated a limited use of conventional drugs in malaria treatment. The common drugs used were paracetamol and aspirin, and they were used with the intention of reducing body temperature of the febrile child. One key important explanation for the limited use of conventional drugs given was it would go against their healing taboos, which they considered cultural sensitive. One traditional healer from Handeni expressed it this way:

In treating my patients, *"I abide to the guide from my ancestral spirits who direct the kind of medication to use. Therefore, I can't use conventional drugs "dawa ya vidonge" because my spirits will ask me where I got them from!"*

When the data was disaggregated on the use of convectional drugs versus level of education, it appeared that the few healers using drugs have had a formal education of standard four to secondary education, and those not using drugs had no formal education.

##### 5^th ^Stage: Prevention of 'degedege'

This important final stage was done with the objective of preventing the child from getting '*degedege*' again and is commonly called '*kufunga*.' Two forms were identified, the first one involved slaughtering a chicken '*kikuku*', a soup which was drunk by the mother and the child to cast out bad spirits '*mashetani*' believed to cause misfortunes to the families. The remains of the chicken were buried a few meters from healers' compound yard and, after the burial, all people involved were required to move while looking forward, no one is allowed to look backward, so as not to get again the devil spirit that caused '*degedege*'.

The last and final form is when a child is tied with amulets in a form of a black piece of cloth '*hirizi*' usually put in the left hand or in the neck as a symbol that the evil spirit '*jini*' was barred from bringing back '*degedege*' to the child. It appeared that most healers indicated to have the powers to protect their clients' children from getting '*degedege*' by preparing charms or amulets or placing 'medicine' on the ground of homestead to contravene the evil effects which was commonly called "*kutoa zongo*" by the Zigua of Handeni District.

It is important to note that, most healers admitted that children do get '*degedege*' in their area because the evil spirits were not cast out of them at birth. One male healer, aged 67, in Kilosa, said he had been called in many homes with new babies to tie a piece of cloth to prevent '*degedege*', thus underscoring the importance of traditional knowledge. It may seem, therefore, that those mothers whose children suffer from degedege, did not get their children tied with the cloth when they were born, hence developed '*degedege*'.

It seems that the healing process is organized in stages and failure to abide '*kinyume cha taratibu*' to the procedure may lead to relapse of '*degedege*', which was believed to be caused by evil spirits.

#### Referral system

Analysis of findings from traditional healers indicated that there exists a form of referral system for severe cases from one traditional healer to other healers or health facilities.

Over 85% of traditional healers (N = 41) agreed they refer cases of malaria to health facilities. Only 15% of the healers admitted that referrals were done to other healers on conditions such as casting out of demons and changing of the colour of the child, which necessitated referral to nearby facility. It is worth noting the existence of healers who specialized in casting demons- these received clients from other healers in the study area.

It also emerged that referrals to health facilities increased with the introduction of Rectal Artesunate Project, whereby the project aides facilitated referral after provision of a suppository. With regard to who decided on when and where the child is to be referred to, it emerged both from mothers and healers interviewed, that the mothers would consult the healer and that a mutual agreement was reached. In a situation where there was no mutual agreement, it sometimes happened that the mother would take the decision herself and take her child to the health facility. Close to a three quarter of mothers interviewed said that they would prefer referral to a heath facility, compared to referral to another healer, in a situation of worsening condition of the child. It has to be noted that the referral to the facility did not indicate that the mother would not come back to the healer again – there was movement from the healer to health facility, back and forth, even when the condition of the baby showed improvement. Revisiting healers was mainly done with the purpose of cleansing the child from contracting '*degedege*' again. A study in Kilombero district in Tanzania [20] concluded that decisions to revisit healers '*waganga*' was taken after several visits to hospitals and because they were sceptical about having received the correct examination or the correct treatment.

#### Collaboration of traditional healers and Rectal Artesunate Project

All the traditional healers interviewed (N = 41) said they were aware of the Rectal Artesunate Project and admitted to have collaborated with the project staff in one way or another since 2000. Project staff constantly visited the healers and, in collaboration with project aides recruited in the areas, they helped manage severe cases of malaria. It also emerged that the project aides were well known and respected persons in their communities and many women with children suspected with malaria turned to them, either directly or through healers.

However, some healers complained of project aides robbing them of their clients who used to visit them: these now go to the project aides directly, thereby bypassing the healers. In addition, the healers felt that the project aides had more recognition (for example, identification on Identity Cards) and monetary motivation (salaries/allowances). They requested for some form of recognition, as the project gets some of its clients through them. Majority of the healers (85%) were willing to collaborate with the Rectal Artesunate Project in reducing morbidity and mortality of malaria among children in the studied areas. They identified possible ways to enhance the existing collaboration (Table 2).

**Table 2:** Collaboration of Traditional healers

**Area of Collaboration**	**% Agreement**
More research on local herbs used to treat malaria	40%
Joint training on how to manage severe cases between healers and health workers	85%
Increased – respect by health workers to traditional healers	80%
Provision of Identity Cards (IDs) just as those project aides who wear IDs claiming that they are all service providers and the society recognizes them	35%

## Discussion

Qualitative data have indicated that people in the study areas have integrated conceptions derived from biomedicine into their local knowledge and practice. It is evident that most of interviewed mothers possess a good knowledge in recognizing mild malaria, which is consistent with the biomedical definition of mild malaria. Most mothers perceived *degedege *(convulsions) as a serious childhood condition, caused by evil spirits, which attacks a child in conjunction with malaria. Mothers related mild malaria to mosquito bites, whilst *degedege*, though acknowledged as being a serious childhood condition, was not recognized as possible outcome of malaria infection. The local interpretation of malaria and related complications are reflected to the logic of actions and treatment-seeking behaviours followed for a sick child. In view of the fact that severe forms of malaria are linked to supernatural causes, a traditional healer is considered more appropriate for treating a convulsing child. Similar findings have been reported elsewhere [21] whereby, even if mothers of under-five children are able to provide descriptions of the condition, including convulsions, there had contradictory views concerning the causes and management of the condition. The relationship between convulsions and evil spirits is a perception that is common to other African communities [8,22,23]. Modern health care was sought for what was perceived to be mild malaria, while traditional care was sought for *degedege*.

Although traditional health practitioners participating in this study were not linking *degedege *to biomedically-defined malaria, they affirmed that, when convulsion occurs in a child, malaria is its usual companion. On this realization, they refer their patients to health facilities for malaria treatment immediately after arresting the spirits causing the convulsion. Furthermore, with the introduction of the Rectal Artesunate Project in the study areas, traditional health practitioners were provided with a nearby health service for referring their patients, hence an increase of the number of referrals from traditional healers. None of the other studies on *degedege *[7,8,23,24] had exposed clearly the facilitating role of traditional healers in the management of severe malaria in children. The observed increased referrals to the Rectal Artesunate Project centres for modern medicine after traditional care is a significant cooperation between traditional healers and the Rectal Artesunate Project centres which are close-by.

There are healers in almost every village and they represent the most numerous health care providers in Tanzania, with an estimated 1: 350 healer per population [25], as compared to 1:33,000 medical doctor per population. Indeed, healers are respected by the community, partly because of their acquired knowledge, their age, their kindness (warm welcome during consultation), their ability to provide answers and treatments that are meaningful to the community.

Some of the barriers to smooth collaboration between biomedical health personnel and healers include:

• Natural competition for prestige and patients

Reluctance of biomedical personnel to cooperate with healers, because of a genuine concern that their practice may harm the patients, which may not always be true.

• Negative attitude by most biomedical towards traditional health practices, by branding it as witchcraft.

• Government officials and biomedical staff having little or no knowledge of the actual healing practices of traditional healers.

One of the key recommendations from this study is to enhance collaboration by focusing on improving the capacity of traditional healers to assist their patients on referral and on decreasing possibly harmful traditional practices. More operational research is needed to clarify the best approaches to make such a collaboration possible.

## Conclusion

The introduction of Rectal Artesunate Project in the study areas is a positive step towards collaboration between modern and traditional health practices in the management of malaria in children. The collaboration in the Rectal Artesunate Project area has convinced the community that dual health services are possible and represent a positive step towards early referral and prompt treatment, hence, reducing morbidity and mortality. The involvement of traditional healers has improved management of severe malaria by introducing the new paradigm that, after convulsions in a child, medical care for malaria is necessary, before convulsion-preventive rituals can be carried out. The introduction of artesunate suppositories was acceptable to traditional healers and improved referrals. The study has gone some way to remove the blame from traditional healers accused in delaying access of sick children to prompt medical care.

## Authors' contributions

E A Makundi obtained funding, contributed to the study conception, design, fieldwork, data analysis, interpretation and drafting, revision and final approval of the manuscript. H Malebo contributed to data analysis, data interpretation, revision and final approval of the manuscript. P Mhame participated in data collection, data analysis, reviewed draft of the report and approved the final version. A Y Kitua contributed to study conception, design, drafting the first version, revision and approval of final version. M Warsame obtained funding, contributed to the study conception, design, data interpretation and drafting, revision and final approval of the manuscript.

## Conflict of interest statement

The author(s) declare that they have no competing interests.
